# Potentiating response of D- Ribose-L-Cysteine on Sodium arsenate- induced hormonal imbalance, spermatogenesis impairments and histomorphometric alterations in adult male Wistar rat

**DOI:** 10.5935/1518-0557.20200109

**Published:** 2021

**Authors:** B. Ogunlade, S. A. Adelakun, V.O. Ukwenya, T.T. Elemoso

**Affiliations:** 1 Human Anatomy Department, Federal University of Technology Akure, Ondo State

**Keywords:** Sodium Arsenate, D-Ribose-L-Cysteine, Semen parameters, Oxidative stress, and Testicular histomorphometry

## Abstract

**Objective:**

Reproductive toxicity is an important health challenge, mostly associated with exposure to several environmental toxicants. Arsenic is a ubiquitous toxic compound naturally present in the environment. This study was carried out to evaluate the dietary supplements of D-Ribose-L-Cysteine against sodium arsenate-induced testicular toxicity in adult male Wistar rats.

**Methods:**

A total of 32 male rats (150-250g) were randomly divided into four (4) groups (n=8). Group A received normal saline as placebo; Group B received 8mg/kg BW of Sodium arsenate only; Group C received 8mg/kg BW of Sodium arsenate and 10 mg/kg BW of D-Ribose- L-cysteine; Group D received 8mg/kg BW of Sodium arsenate and 30 mg/kg BW of D-Ribose- L-cysteine. All administration was done via oral gavage for 28 days, thereafter the animals were sedated with pentobarbital sodium (intraperitoneally); we obtained testes and blood serum for analysis.

**Results:**

The results showed abnormal testicular morphology with degeneration and decrease in spermatogonia, vacuolation and empty lumen, intense necrosis, spermatogenesis disruption (decrease sperm count, motility, viability) and degraded germinal epithelium of the seminiferous tubules, reduction in the hormone profile (FSH, LH, and TT) and oxidative stress parameters (CAT, GSH, and SOD) with a corresponding increase in MDA level in the arsenic-only treated rats (group B) compared to their control counterparts (group A), but it was ameliorated after DRLC administration, both in low and high doses, respectively.

**Conclusions:**

D-Ribose-L-Cysteine attenuated distorted testicular morphology, altered semen characteristics, hormone profile, and oxidative stress markers by preventing the deleterious toxicity of sodium arsenate.

## INTRODUCTION

Exposure to heavy metals has been shown to pose serious health hazards, causing neurotoxicity, genotoxicity, and carcinogenicity induced primarily by oxidative stress and high affinity for active functional proteins (thiol groups) (Hultberg *et al.,* 2001; Flora *et al.,* 2007). Arsenic is a toxic metalloid present in the environment in organic or inorganic states. It is predominantly abundant in surface and groundwater reservoirs, and greatly solubilized depending on the PH, redox conditions, solution composition, and temperature (Otleş & Cağindi, 2010). There are several sources of man-made arsenic contaminating soil and drinking water, which includes mineral extraction and processing wastes, poultry and swine feed additives, pesticides, and highly soluble arsenic trioxide stockpiles, that have adverse consequences on multiple organ systems (Nordstrom, 2002; Otleş & Cağindi, 2010). Sodium arsenate is an inorganic source of arsenic, attributed to a wide distribution and adverse health effects, regarded as the most dangerous poisons causing major health problems worldwide (Hopenhayn-Rich *et al.,* 1999). Arsenic-contaminated water has been implicated to cause several health conditions including hypertension, diabetes mellitus, human skin, kidney, lung, and bladder cancer (Chen *et al.,* 1988;Ratnaike, 2003; Singh *et al.,* 2011), black foot disease, nervous system disorders, skin lesions, cardiovascular diseases (States *et al.,* 2009), and respiratory diseases (Parvez *et al.,* 2010). The toxicity associated with Arsenic was reported to involve numerous inhibiting enzymes attributed to cellular energy pathways, repair and synthesis of DNA, that is metabolized by reduction and methylation reactions (Ratnaike, 2003; Singh *et al.,* 2011). In addition, environmental sodium arsenate is a common toxin, capable of suppressing spermatogenesis and androgenesis, thereby causing reduced and abnormal sperm production associated with male infertility (Rosenblatt & Burnstein, 2009), and several morphological modifications of the testis such as impaired and apoptotic germ cells, and necrotic seminiferous tubules (Hopenhayn-Rich *et al.,* 1999; Marettová *et al.,* 2014). Increased environmental toxicants result in the build-up of Reactive Oxygen Species (ROS) in the cells, thereby causing an imbalance in the free radicals and antioxidants defense mechanisms of the body (Marettová *et al.,* 2014). Oxidative stress is usually associated with an increased level of free radicals attributed to a decrease in antioxidant defense systems within the body, such as glutathione (Gaballa *et al*., 2010). Also, oxidative stress has been implicated in testicular dysfunctions induced by xenobiotics, thereby contributing to male infertility.

D-Ribose-L-Cysteine (DRLC) is a synthetic supplement that increases the glutathione levels in the body cell for adenosine triphosphate production (Falana *et al.,* 2017). The ribose sugar present in DRLC plays a vital role by protecting cysteine from the enzymatic reactions when administered orally (Nagasawa, 2013). Due to the increased level of free radicals in the body, potent synthetic antioxidants that help cells produce glutathione are usually required to compliment the levels of antioxidants in the body, thereby enhancing the free radical scavengers in the body (Falana *et al.,* 2017). Glutathione is an important biological system (antioxidants) capable of scavenging free radicals/ROS implicated in causing damage to body tissues (Chandra *et al.,* 2015). Therefore, this study aimed at investigating the antioxidant potential of DRLC in sodium arsenate-induced testicular toxicity in adult male Wistar rats.

## MATERIALS AND METHODS

### Chemicals

Sodium arsenate was procured from Sigma Company (St. Louis, MO, USA) and D-Ribose-L-Cysteine (DRLC) was procured from Max International, Salt Lake, USA. All other chemicals used in the study were of analytical reagent grade. 

### Animal procurement

Thirty-two (32) adult male Wistar rats weighing 150-200g (aged 8-10 weeks) were obtained from the breeding stock, at the Federal University of Technology, Akure. The rats were collected in the isolated cages and acclimatized for 7 days in the experimental house of the Department of Human Anatomy, Federal University of Technology, Akure before the commencement of the experiment. They were maintained in constant 12h/12h dark and light cycles at room temperature. The processes of protocols using the experimental animals followed the Guide for the Care and Use of Laboratory Animals (National Research Council (US) Committee for the Update of the Guide for the Care and Use of Laboratory Animals, 2011).

### Experimental protocol

The rats were divided into four groups (n = 8), labeled groups A, B, C, and D. The Arsenic (Mehranjani & Hemadi, 2007) and DRLC dosages (Osinubi *et al.,* 2018) were prepared daily. Group A received normal saline as *placebo*; Group B was administered with 8mg/kg BW arsenic only; Group C was administered with 8 mg/kg BW arsenic followed by 10 mg/kg BW DRLC (low dose) and Group D were administered with 8mg/kg BW arsenic followed by 30 mg/kg BW DRLC (high dose). The administration was done once daily through oral gavage for 28 days after which the animals were slaughtered (Adelakun *et al.,* 2019). All animals were observed for any anomalies, illnesses, and physical anomalies. The experimental procedures followed the recommendations provided in the “Guide for the Care and Use of Laboratory Animals” prepared by the National Research Council (US) Committee for the Update of the Guide for the Care and Use of Laboratory Animals (2011). The rats were fed with standard rat chow, and drinking water was supplied *ad libitum*. The weights of the animals were recorded at procurement, during acclimatization, at the commencement of the experiment, and after the completion of the experimental period using a CAMRY^®^ electronic scale (EK5055, Indian).

### Surgical procedure

After the experiment completion, the animals were administered intraperitoneal sodium pentobarbital (40mg/kg) (Adelakun *et al.,* 2019), their abdomens were dissected and their testes were immediately extracted. The serum collected from the blood samples were later centrifuged for analysis.

### Gross observation of the testes

The testes were initially dissected out via midline abdominal incision and cleared of fats and blotted dry. Their weights were measured on a sensitive digital scale with volume measured by water displacement, using a 10-ml measuring cylinder (Acott et al., 1999), as per described by Scherle (1970). Then their sizes (length and width) were recorded using a sliding gauge (d=0.1). Two testes from each rat were measured and the average value obtained for each of the parameters was regarded as one observation. Eventually, they were fixed in freshly prepared Bouin‘s fluid)(Russell *et al*., 1990; Simas *et al.,* 2017).

### Sperm analysis

The spermatozoa from the cauda epididymis were obtained by cutting into 2ml of medium {Hams F10} containing 0.5% bovine serum albumin (Feng *et al.,* 2001) and incubated at 37ºC (with 5% CO_2_) for 5 minutes.

### Sperm motility (motile sperm percentage)

About 5.0 µL of supernatant containing sperm was placed between the slide and the coverslip, and observed at 100 × magnification using a light microscope (Leica DM750). The sperm movement evaluation was held in three different fields, and motility was expressed from the middle of the fields in the percentage of motile sperm from the total sperm counted (Badkoobeh *et al.,* 2013).

### Sperm count (×10^6^sperm/mL)

About 10 µL of the supernatant containing the epididymal sperm were diluted in 990 µL of a paraformaldehyde and sodium citrate solution. Approximately 10 µL of diluted content were transferred to a hemocytometer (Neubauer chamber) (Deep1/10mm LABART, Germany), which was assessed under light microscopy at 400×. The pelleted cells were counted on the surface of the chamber. We calculated sperm concentration according to the number of cells counted and hemocytometer dimensions. The concentration was expressed in millions of sperm per mL (Badkoobeh *et al.,* 2013).

### Sperm morphology (percentage of normal cells)

To analyze sperm morphology we placed a drop of about 20 µL of sperm suspension on the microscope slides and swiped. The slides were dried and stained with eosin-nigrosin (1% eosin Y and 5% nigrosine). After drying, they were analyzed under the light microscope (Leica DM750) at 400×. A differential count of 200 spermatozoa per slide was performed and we looked for changes to the head, middle piece, and tail. The results were expressed as the percentage of normal cells (Badkoobeh *et al.,* 2013).

### Sperm viability

An aliquot of 20 µL of the suspension containing the sperm was diluted with an equal volume of nigrosine-eosin (1% eosin Y and 5% nigrosine). Then, a smear of content was carried out on the microscope slide and after drying, the preparations were evaluated under light microscopy (Leica DM750) at 400×. We performed the differential count of 200 spermatozoa by observing the proportion of unstained sperm (full membrane, said as viable) on the colored (non-intact membrane, said as non-viable). The results were expressed as the percentage of spermatozoa with the intact membrane (viable) on total sperm count (Badkoobeh *et al.,* 2013).

### Histomorphometric assessments of the Seminiferous Tubules

The histology slides were prepared from testes fixed in Bouin’s fluid. Before embedding, the sections were placed perpendicular to their long axes and chosen as the vertical section. Five vertical sections from polar and equatorial regions were sampled for each testis (Qin & Lung, 2002). The images were captured using the 10x objective lens from each animal tissue at 15 different fields, and analyzed with the ImageJ software (NIH).

The average tube diameter and the height of the seminiferous epithelium were obtained for each animal from the measurement of 30 cross-sections of seminiferous tubule contours as circular as possible, not taking into account the stage of the seminiferous epithelium cycle (Berndtson *et al.,* 1989). The total length of the seminiferous tubules was estimated from the volume occupied by the seminiferous tubules in the testes, and we calculated the mean tubular diameter obtained for each animal, using the formula CT=VTS/πR^2^(VTS = total volume of seminiferous tubules; πR^2^ = cross-sectional area of the seminiferous tubules; R = tubular radius) (Attal & Courot, 1963). The number of Leydig cells per testis was estimated from the individual volume of Leydig cells and the volume occupied by the Leydig cells in the testis.

### Biochemical Analysis

#### Lipid peroxidation assay

The lipid peroxidation assay was carried out according to the modified method of Ohakawa *et al.* (1979). Briefly, 300 ml of the homogenate was added to 300 ml of 8.1% sodium dodecyl sulfate (SDS), 500 ml of acetic acid/HCl buffer (pH 3.4), and 500 ml of 0.8% thiobarbituric acid (TBA). This mixture was incubated at 100ºC for 1h, and the thiobarbituric acid reactive species (TBARS) produced were measured at 532 nm using a spectrophotometer. Malondialdehyde (MDA) was used as standard, and the TBARS produced was reported as MDA equivalent. MDA was expressed in µmol/mg of protein

#### Superoxide dismutase (SOD) assay

SOD activity was determined by the method of Alia *et al.* (2003), in which 0.05 ml of tissue homogenate was treated with 1.0 ml of 50 mmol/L carbonate buffer (pH 10.2) and 0.017 ml of adrenaline (0.06 mg/ml). The absorbance was read at 480 nm in the spectrophotometer for 2 min at 15-s intervals. SOD activity was expressed in µ/mg of protein.

#### Reduced glutathione assay (GSH)

The GSH content was determined by the modified method of Ellman (1959). One milliliter of the supernatant was added to 0.5 ml of Ellman’s reagent (19.8 mg of 5,5′ dithiobisnitrobenzoic acid in 100 ml of 0.1% sodium citrate) and 3.0 ml of 0.2 mol/L phosphate buffer (pH 8.0). The absorbance was read at 412 nm in a spectrophotometer. GSH was expressed in µmol/mg of protein.

#### Catalase (CAT) assay

The catalase extraction assay was prepared by homogenizing fresh samples (200 mg) in 5 ml of 50 mM Tris-NaOH at pH 8.0, that contained 0.5% (v/v) Triton X-100, 2% (w/v) PVP, and 0.5 mM EDTA. The homogenate was centrifuged for 10 min at 4ºC at 22,000 × g and the resultant supernatant was dialyzed before the enzyme assay. The catalase assay was conducted by adhering to the method suggested by Clairborne (1985). 1ml of reaction mixture containing 50 mM of potassium phosphate buffer (pH 7.0) and 250 µl of enzyme extract was initiated by adding 60 mM of hydrogen peroxide. We measured the absorbance using a spectrophotometer at an absorbance rate of 240 nm for 3 min. The H_2_O_2_ decomposition is calculated by using an extinction coefficient of 39.4 mM^−1^ cm^−1^. The values were expressed as mmol of H_2_O_2_/mg protein/min.

### Hormone Assessment

The hormonal levels of testosterone (TT), Follicle Stimulating Hormone (FSH) and Luteinizing Hormone (LH) were measured using the available immunoassay (ELISA) kits (Randox Laboratories Ltd, Admore Diamond Road, Crumlin, Co., Antrim, United Kingdom, Qt94QY), according to the manufacturer’s instructions (Adelakun *et al.,* 2019).

### Cell counts and cell numbers

The spermatogenesis assessments were stereologically evaluated to ascertain its efficiency according to the method by Russell *et al.* (1990) and Petersen & Pakkenberg (2000). 50 seminiferous tubules selected from each group were evaluated for spermatogonia, spermatocytes, and spermatids. The Sertoli cell counts and the Leydig cell numbers were evaluated in the interstitial tissue. The average of the different cell counts from each animal was used for the analysis. The evaluations of all samples were performed at a constant magnification of 40X with light microscopy (Olympus, Japan).

### Testes Histomorphology

The testes of the rats were removed and fixed in Bouin‘s fluid for 24h and underwent tissue processing as described by Leblond & Clermont (1952). The slides were then stained with Hematoxylin and Eosin, mounted in DPX, and the photomicrographs were taken at a magnification of 400× on a Leica DM750 microscope.

### Data presentation and statistical analysis

The data obtained were statistically analyzed using the one-way ANOVA with GraphPad Prism 5 Windows software, followed by Dunnett’s comparison test. The data were expressed as Mean±SEM. The level of significance was set at *p*<0.05.

## RESULTS

### EFFECT OF DRLC ON BODY AND TESTICULAR WEIGHT IN ARSENIC-INDUCED NORMAL AND EXPERIMENTAL RATS

**Figure 1:**
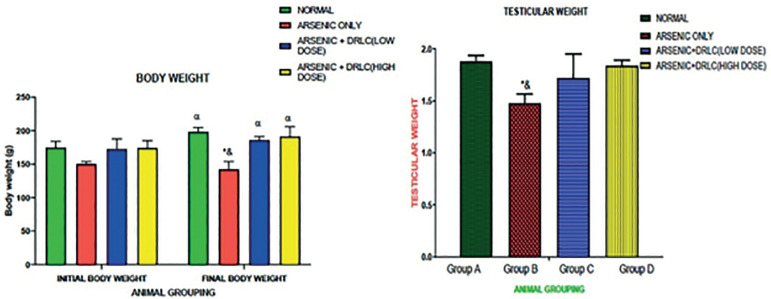
DRLC effect on the body weight and testicular weight on Arsenic-induced normal and experimental rats. *: *p*<0.05 as compared to group A; α: *p*<0.05 as compared to group A, C and D; &: *p*<0.05 as compared to group C and D.

The result revealed that rats treated with Arsenic only (Group B) had a significant decrease (*p*<0.05) in body (final) and testicular weight when compared to the control animals (group A) (Fig. 1). However, the rats administered with both Arsenic and DRLC in high and low doses (Groups C and D) showed no significant difference when compared with the control animals (Group A) but there was a significant increase in testicular weight (*p*<0.05) when compared with the Arsenic only group (Group B) (Fig. 1).

### DRLC EFFECTS ON the STEREOLOGICAL EVALUATIONS IN SODIUM ARSENATE-INDUCED TESTICULAR TOXICITY-TREATED RATS

The result showed a significant decrease (*p*<0.05) in testicular diameter (TD), testicular volume (TV), testicular length (TL), seminiferous tubules diameter (STD), spermatogonia nuclear diameter (NDS) and germinal thickness of epithelium (GTE) with a corresponding increase in lumen diameter of tubules (LDT) among the rats treated with Arsenic only (Group B) when compared to the controls (Group A) (Table 1). However, the rats administered with both arsenic and DRLC in high and low doses (Groups C and D) showed a significant increase (*p*<0.05) in TD, TV, TL, STD, NDS, and GTE with the corresponding decrease in LDT, when compared with the arsenic only group (Group B) (Table 1). In addition, there was a significant decrease in TD, TV, TL, STD, NDS, and GTE when the rats administered with both arsenic and DRLC in high and low doses (Groups C and D) were compared with the controls (Group A) (Table 1), except in LDT that exhibited significant increases (*p*<0.05) (Table 1).

**Table 1:** DRLC responses on testicular diameter, testicular volume, testicular length, seminiferous tubule diameter, lumen diameter of tubules, nuclear diameter of spermatogonia and germinal thickness of epithelium in sodium arsenate-induced testicular toxicity treated rats.

Parameters	Animal Grouping			
	Group A	Group B	Group C	Group D
Testicular diameter (mm)	12.12±2.35	4.73±2.14*^&^	8.28±1.05^#^	9.25±1.12^#^
Testicular volume (cm3)	2.7±0.5	1.6±0.3*^&^	2.1±0.7^#^	2.2±0.4^#^
Testicular Length (mm)	23.46±2.51	14.25±3.18*^&^	18.05±2.15^#^	20.11±2.16^#^
Diameter of ST (µm)	173.08±7.34	145.51±4.20*^&^	163.22±3.63^#^	167.15±4.18^#^
Lumen diameter of tubules (µm)	70.14±3.52	95.49±5.23*^&^	80.08±3.51^#^	78.82±2.45^#^
Nuclear diameter of spermatogonia (µm)	4.15±0.24	3.95±0.78*	4.1.±0.62	4.05±0.78
Germinal thickness of epithelium (µm)	51.34±2.41	33.71±3.52*^&^	44.23±3.17^#^	46.67±3.21^#^

Data expresses as Mean ± SEM (n=8).

* *p*<0.05 compared with control group;

& *p*<0.05 compare with groups C and D;

# *p*<0.05 compared with control. One-way ANOVA using Dunnett's comparison test.

ST: seminiferous tubule; Group A= Control (Normal saline); Group B= Sodium arsenate only; Group C= Arsenic +DRLC (Low dose); Group D= Arsenic +DRLC (High dose).

Furthermore, the results showed a significant decrease (*p*<0.05) in the number of Sertoli cells, Leydig cells, spermatogonia, spermatogenesis index, spermatocytes and spermatid among the rats treated with Arsenic only (Group B), when compared to controls (group A) (Table 2). However, the rats administered with both arsenic and DRLC in high and low doses (Groups C and D) showed a significant increase (*p*<0.05) in the number of Sertoli cells, Leydig cells, spermatogonia, spermatogenesis index, spermatocytes and spermatids, when compared with the arsenic-only group (Group B) (Table 2). Also, there was a significant decrease in the number of Sertoli cells, Leydig cells, spermatogonia, spermatogenesis index, spermatocytes, and spermatid when the rats treated with both arsenic and DRLC in high and low doses (Groups C and D) were compared with the controls (Group A) (Table 2).

**Table 2:** DRLC responses on Sertoli cell, Leydig cell, spermatogonia, spermatogenesis index, spermatocytes and spermatid in sodium arsenate induced testicular toxicity treated rats.

	Sertoli cell	Leydig cell	Spermatogonia	Spermatogenesis index	spermatocytes	spermatids
Group A	37.12±4.15	15.75±2.05	8.05±1.28	105.25±8.45	25.34±3.08	95.65±5.64
Group B	15.17±2.14*^&^	6.22±1.17*^&^	3.15±0.92*^&^	55.32±5.71*^&^	12.43±3.27*^&^	35.72±7.29*^&^
Group C	29.43±2.18^#^	10.35±2.11^#^	5.51±1.22^#^	78.05±7.28^#^	19.58±2.41^#^	78.53±5.28^#^
Group D	32.05±3.67^#^	12.51±1.89^#^	6.05±1.75^#^	83.72±5.25^#^	22.25±2.18^#^	82.36±4.25^#^

Data expresses as Mean±SEM (n=8).

* *p*<0.05 compared with control group; &:*p*<0.05 compare with groups C and D;

# *p*<0.05 compared with control. One-way ANOVA using Dunnett's comparison test. Group A= Control (Normal saline); Group B= Sodium arsenate only; Group C= Arsenic +DRLC (Low dose); Group D= Arsenic +DRLC (High dose)

### DRLC EFFECTS ON SPERM ANALYSIS IN ARSENIC-INDUCED NORMAL AND EXPERIMENTAL RATS

**Figure 2:**
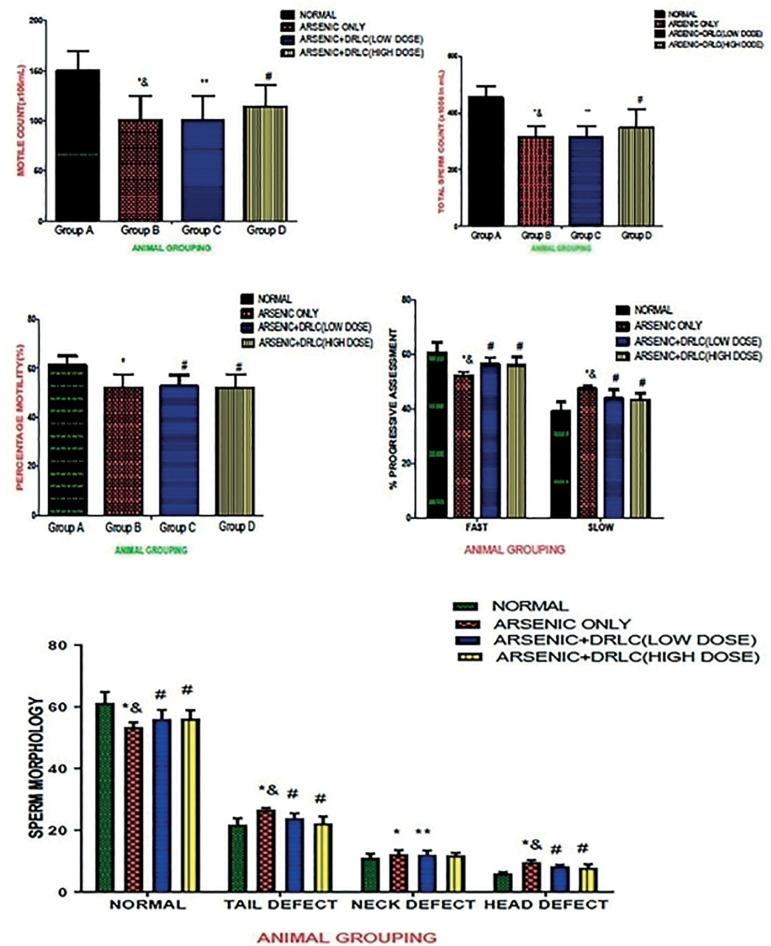
Effect of DRLC on sperm parameters on Arsenic-induced normal and experimental rats. *: *p*<0.05 as compared to group A; **: *p*<0.05 as compared to group A and D; &: *p*<0.05 as compared to group C; #: p<0.05 as compared to groups A and D.

The results revealed that rats treated with Arsenic only (Group B) showed a significant decrease (*p*<0.05) in concentration, count, percentage, motility and sperm viability with a corresponding increase in sperm defects when compared to the controls (Group A) (Fig. 2). However, the rats administered with both arsenic and DRLC in low and high doses (Groups C and D) showed a significant increase in concentration, count, percentage, motility, sperm viability with the corresponding decrease in sperm defects when compared with the Arsenic-only group (Group B) (*p*<0.05) (Fig.2).

### EFFECT OF DRLC ON OXIDATIVE STRESS MARKERS (MDA, GSH, SOD, AND CAT) IN ARSENIC-INDUCED NORMAL AND EXPERIMENTAL RATS

**Figure 3:**
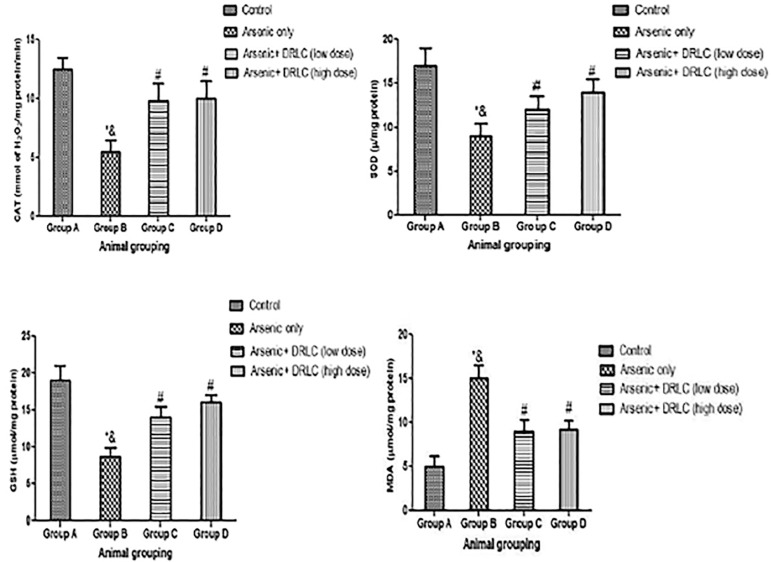
Effect of DRLC on CAT, GSH, SOD and MDA levels of Arsenic-induced normal and experimental rats. *:*p*<0.05 as compared to group A; &:*p*<0.05 as compared to group C and D; #:*p*<0.05 as compared to groups A.

The results revealed that rats treated with arsenic only showed a significant decrease in GSH, SOD, and CAT levels (Fig. 3) with a corresponding increase in MDA (*p*<0.05) (Fig.3) when compared to the control animals (Group A). However, the rats administered with both arsenic and DRLC in high and low doses (Groups C and D) showed a significant increase in GSH, SOD, and CAT activities (Fig. 3), with the corresponding decrease in MDA when compared with the arsenic-only group (Group B).

Also, there was a significant difference in MDA, GSH, SOD, and CAT activities when the rats administered with both arsenic and DRLC (Groups C and D) were compared with the control Group (Group A) (*p*<0.05) (Fig. 3).

### DRLC EFFECT ON SERUM HORMONE PROFILE (FSH, LH, AND TT) IN ARSENIC-INDUCED NORMAL AND EXPERIMENTAL RATS

**Figure 4:**
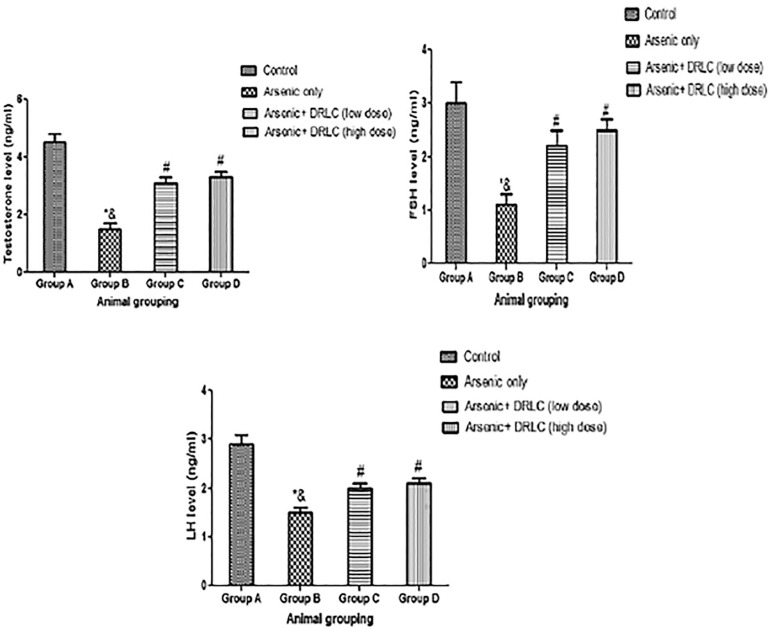
Effect of DRLC on serum level of FSH, LH and TT on Arsenic-induced normal and experimental rats. *:*p*<0.05 as compared to group A; &:*p*<0.05 as compared to group C and D; #:*p*<0.05 as compared to groups A.

The results showed that rats treated with arsenic-only (Group B) showed a significant decrease in FSH, LH, and TT levels compared to the control animals (Group A) (*p*<0.05) (Fig 4). However, the rats administered with both arsenic and DRLC in high and low doses (Groups C and D) showed a significant increase in FSH, LH, and TT levels when compared with the arsenic-only group (Group B) (*p*<0.05) (Fig 4),

Although, there was a significant decrease in FSH, LH, and TT activities when the rats administered with both arsenic and DRLC in high and low doses (Groups C and D) were compared with the control animals (Group A) (*p*<0.05) (Fig 4).

### HISTOLOGICAL OBSERVATIONS

**Figure 5:**
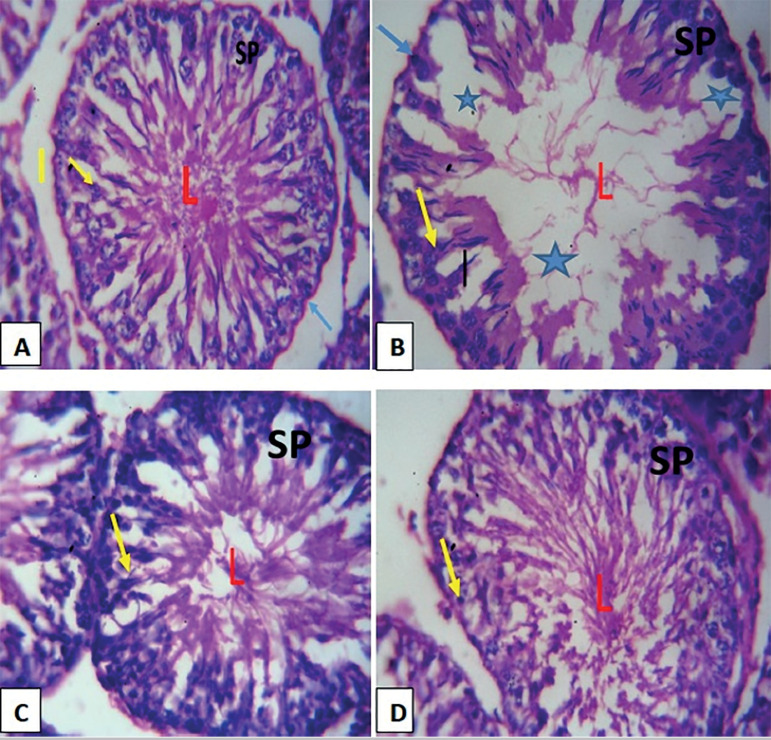
**A:** Group A showing normal histoarchitecture with typically organized layers of spermatogenic cells at different stages of maturation (Yellow arrow), no pathological changes in the lumen (L) of the seminiferous tubules, spermatozoa (SP), basement membrane (blue arrow) and interstitial space (I); **B:** Group B showing testicular photomicrograph section of Arsenic-only group showing distortion of the tubular architecture and disorganization of the spermatogenic cells (yellow arrow) in seminiferous tubules, hypocellularity due to degeneration of germ cells, disruption of spermatogenesis and vacuolation (*) and empty lumen (L); **C:** Group C showing a testicular photomicrograph section of arsenic and DRLC treated group at low dose, showing restored lumen (L) with visible spermatozoa (SP), abundant sperm cells (yellow arrow); **D:** Group D showing testicular photomicrograph section of arsenic and DRLC treated group at high dose, showing a notable restoration of the lumen (L) of the seminiferous tubules, with visible spermatozoa (SP) and abundant sperm cell (yellow arrow) Stains: Hematoxylin and Eosin. Mg X400.

The testicular morphology of the arsenic-only group (Group B) showed necrosis and degeneration, with a decrease in the epithelial layer of germ cells thickness and seminiferous tubules’ diameter, when compared with the control (Group A) (Fig. 5B). Also, the arsenic distorted the seminiferous tubules, with loss of normal distribution of epithelial lining and vacuolar cytoplasm compared with the controls (Group A). However, testicular photomicrograph of the animals that received the combined administration of arsenic with high and low doses of DRLC (Groups C and D) showed improved morphology, with an oval or circular presentation of distinctive stratified seminiferous epithelium, which lumen possesses spermatogenic cells and prominent Leydig cells, similar to the Control Group (Group A).

## DISCUSSION

Reproductive toxicity from heavy metal exposure is a serious medical challenge implicated in male infertility. The present study showed that exposure to Arsenic caused severe testicular toxicity in rats, evidenced by the reduction in the testicular weight/body weight ratio relative to the controls, thereby leading to obstruction of spermatogenesis and steroidogenesis. Our results conform with the reports by Bataineh *et al.* (1998) and Adelakun *et al.*(2019). They deduced that the administration of metals to rats causes a reduction in spermatogenic cell population and a decrease in testicular cell’s nuclei. The decreased in reproductive organ weight found could be attributed to the decrease in hormone levels (especially testosterone) induced by the abundant free radicals within the testes (El-Ashmawy *et al.,* 2007; Sun *et al.,* 2011).

Damage to testicular structural function has been linked to recurrent exposure to oxidative stress, through the involvement of lipid peroxidation as measured by the level of Malondialdehyde (MDA) (Watanabe *et al.,* 2002). In the present study, there was a significant increase in MDA level after sodium arsenate administration, when compared to control animals, suggesting increased production of free radicals, thereby causing damage and loss of functional properties. Tissue levels of MDA is a proven indicator of oxidative stress resulting from lipid peroxidation (Wiseman & Halliwell, 1996; Reiter *et al.,* 2009). However, there was a significant decrease MDA levels in the treated group that received DRLC (both low and high doses), compared to the arsenic-only group, thereby ameliorating the toxic effects of sodium arsenate- induced oxidative stress.

Glutathione (GSH) is an important antioxidant that protects the body against damage to cellular structures caused by the build-up of free radicals or excess ROS (Jurczuk *et al.,* 2006). In the present study, there was a decrease in the levels of GSH, CAT, and SOD in the sodium arsenate-only Group when compared to controls, thereby suggesting the inability of testicular cells to scavenge free radicals (Oyeyipo *et al.,* 2018). This observation is following previous studies that deduced a decreased level of CAT and a corresponding decrease in enzyme activity of free radicals, thereby causing an imbalance against the defense system, leading to morphological disruption and loss of testicular function (Oyeyipo *et al.,* 2018). Also, the increase in SOD activity may have been a result of a *de novo* decreased synthesis of enzyme proteins or inactivation of these enzyme proteins, leading to decreased testicular function (Oyeyipo *et al.,* 2018).However, there was a significant increase in CAT, SOD, and GSH levels, in treated groups that received DRLC (low and high doses) compared to arsenic-only animals, suggesting the ameliorative and antioxidant properties of DRLC (Malhotra & Devi, 2005).

Furthermore, significant decreases in sperm parameters, found in the Arsenic-only group compared to controls was caused by oxidative stress in the tissue characterized by an increase in ROS, leading to an imbalance between the antioxidants in the body tissue and free radicals. This imbalance is a characteristic function of oxidative stress, and it is involved in xenobiotic-induced testicular dysfunctions, which cause male infertility. The significant increase in sperm parameters observed after DRLC (low and high doses) administration was as a result of increased DRLC antioxidant potency. It is believed that the consumption of certain vitamins may help improve male fertility (Malhotra & Devi, 2005).

Spermatogenesis has been an important process occurring in the testis, requiring two main hormones namely LH and FSH. Reports have shown that there is an increase in the spermatogenic potency of the testis when FSH acts on the Sertoli cell (Ogedengbe *et al.,* 2018). In this study, there was a significant decrease in FSH, LH, and TT levels in the Arsenic-only group compared to controls. This finding is following previous reports that showed a reduction in strength and function of spermatogenic cells involved in spermatogenesis, due to declined FSH, LH, and TT levels (Ogedengbe *et al.,* 2018), accompanied by deterioration of Sertoli and Leydig cell function after sodium arsenate. However, there was a significant increase in FSH, LH, and TT in the treated group that received DRLC (low and high doses), compared with Sodium Arsenate only, thereby ameliorating the toxic effects on hormone profile in sodium arsenate-induced testicular toxicity.

Histological investigations revealed nearly organized layers of spermatogenic cells at different stages of maturation, intact lumen of the seminiferous tubules, and spermatozoa after DRLC administration, both in low doses and high doses compared with arsenic-only, which showed distortion of the tubular architecture and disorganization of the spermatogenic cells in seminiferous tubules, hypocellularity due to degeneration of germ cells, disruption of spermatogenesis and empty lumen. The testicular structural disorganization after arsenic administration found corroborates previous studies on the toxic effect of metals, such as aluminum, sodium fluoride, lead, nickel reported to cause derangement of testicular morphology and functions (Chinoy *et al.,* 2005; Hala *et al.,* 2010). The amelioration of testicular morphology found could be attributed to the DRLC’s ability to boost the body`s antioxidant status against the deleterious insult of ROS, thereby restoring the lumen of the seminiferous tubules with visible spermatozoa and abundant sperm cell.

## CONCLUSION

D-Ribose-L-cysteine plays a protective role against arsenic-induced testicular disruption of the morphology and homeostasis of the testis. This study further ascertains fertility-enhancing capacity and the antioxidant properties of D-Ribose-L-cysteine in maintaining testicular functional and structural integrity.
